# Supplementary material for: Health-Related Quality of Life (HRQoL) and the Effect on Outcome in Patients Presenting with Coronary Artery Disease and Treated with Percutaneous Coronary Intervention (PCI): Differences Noted by Sex and Age
Source: J Clin Med. 2022 Sep 5;11(17):5231. doi: 10.3390/jcm11175231 (PMC9457487; doi:10.3390/jcm11175231)
Supplement: Supplementary file 1 [file jcm-11-05231-s001.zip › jcm-1873677-supplementary.pdf]

Supplementary Table S1: Baseline Characteristics of patients by availability of pre-procedure HRQoL.

**Characteristics n (%)**	**QoL info *not* available**	**QoL info available**	**P value**
	8607	6591	
Male	6534 (76.0%)	5068 (76.9%)	0.19
Age, years, mean (SD)	68.4 (10.9) (n=8595)	69.2 (10.0) (n=6583)	<0.001
Diabetes	2110 (24.5%)	1587 (24.1%)	0.53
Hypertension	6238 (72.5%)	4760 (72.2%)	0.73
Family History of CAD	3228 (37.5%)	2214 (33.6%)	<0.001
Smoking (past or current)	4335 (50.4%)	3550 (53.9%)	<0.001
BMI, kg/m^2 ±SD	28.8 (5.0) (n=7762)	29.0 (5.0) (n=6109)	0.03
LVEF, mean ± SD	57.0 (10.6) (n=6829)	56.7 (9.4) (n=5650)	0.17
Previous MI	1910 (22.2%)	1425 (21.6%)	0.40
Previous Peripheral Vascular Disease	641 (7.4%)	467 (7.1%)	0.39
Previous PCI	2841 (33.0%)	2077 (31.5%)	0.05
Previous Cerebrovascular disease	579 (6.7%)	450 (6.8%)	0.81
Previous CABG	933 (10.8%)	702 (10.7%)	0.71
Previous HF	466 (5.4%)	313 (4.7%)	0.07
Current HF (<2 wks)	350 (4.1%)	190 (2.9%)	<0.001
Renal failure	485 (5.6%)	314 (4.8%)	0.01
**Clinical presentation**			
STEMI	686 (8.4%)	374 (5.8%)	<0.001
NSTEMI	1847 (22.7%)	1408 (21.7%)	0.16
Unstable angina	1331 (16.3%)	1025 (15.8%)	0.38
Elective	4278 (52.5%)	3675 (56.7%)	<0.001
Cardiogenic Shock	54 (0.6%)	15 (0.2%)	<0.001

CAD-Coronary artery disease; BMI-Body mass index; LVEF-Left ventricular ejection fraction; CABG-Coronary artery bypass grafting; MI-Myocardial infarction; HF-Heart failure; STEMI-ST-elevation myocardial infarction; NSTEMI-Non-ST-elevated myocardial infarction; SD-Standard deviation.

Renal failure/impairment is defined as either (a) Sr. Creatinine >2mg/dl and/or (b) having renal failure/receiving dialysis.

Supplementary Table S2: Pre-procedure HRQoL and its association with mortality and MACE within one-year following procedure in the overall patients and by sex.

**Event**	**Overall**	**Women**	**Men**
	HR (95% CI)	HR (95% CI)	HR (95% CI)
**Mortality**			
Unadjusted	3.09 (1.89-5.06) p<0.001	1.32 (0.57-3.05) p=0.52	4.72 (2.52-8.84) p<0.001
Adjusted*	2.41 (1.44-4.02) p=0.001	1.02 (0.41-2.54) p=0.97	3.99 (2.09-7.65) p<0.001
**MACE**			
Unadjusted	1.36 (1.06-1.74) p=0.02	1.31 (0.81-2.10) p=0.27	1.38 (1.03-1.84) p=0.03
Adjusted*	1.31 (1.02-1.69) p=0.04	1.27 (0.78-2.08) p=0.34	1.31 (0.97-1.77) p=0.07

* Adjusted for age, male (except for gender specific analysis), BMI, Smoking ever, PCI presentation, diabetes, LVEF, Previous MI, PVD, previous CABG, HF within prior 2 weeks of PCI, renal failure, cardiogenic shock
